# Di-μ-chlorido-bis­(chlorido­{8-[2-(di­methyl­amino)­ethyl­amino]­quinoline}­cadmium) ethanol monosolvate

**DOI:** 10.1107/S2414314621001504

**Published:** 2021-02-16

**Authors:** Abdul-Razak H. Al-Sudani, Myasim Qasim Abdulridha, Benson M. Kariuki

**Affiliations:** a Department of Chemistry, College of Science for Women, University of Baghdad, Iraq; b School of Chemistry, Cardiff University, Main Building, Park Place, Cardiff CF10, 3AT, UK; University of Aberdeen, Scotland

**Keywords:** crystal structure, cadmium, complex, quinoline

## Abstract

The title solvated bimetallic complex, [Cd_2_Cl_4_(C_13_H_17_N_3_)_2_]·C_2_H_5_OH, comprises two Cd^2+^ metal ions linked by a pair of μ^2^ Cl^−^ ions. The coordination sphere around each Cd^2+^ ion is completed by three N atoms of a tridentate 8-[2-(di­methyl­amino)­ethyl­amino]quinoline ligand and another chloride ion to form a distorted *fac*-CdN_3_Cl_3_ octa­hedron. The ethanol mol­ecule is both an acceptor of an N—H⋯O and a donor of an O—H⋯Cl hydrogen bonds to its adjacent complex unit. In the crystal, weak aromatic π–π stacking is observed.

## Abstract 2

In the title bimetallic complex, two Cd^2+^ metal ions are linked by a pair of Cl^−^ ions and their coordinations completed by another Cl^−^ ion and three N atoms.

## Graphical abstract

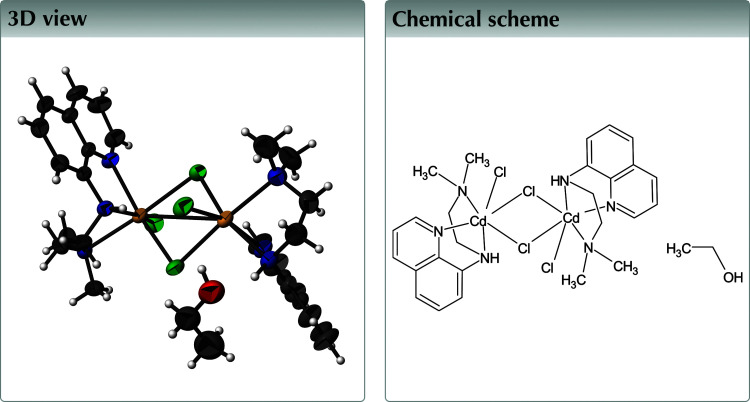

## Structure description

Part of our research in metal coordination chemistry includes the investigation of N-containing ligands with the quinoline moiety (Amoroso *et al.* 2009 ▸; Al-Sudani, 2014 ▸; Kariuki & Al-Sudani, 2014 ▸). The title structure, **I**, is an ethanol solvate of the complex previously obtained in hydrate form (Al-Sudani & Kariuki, 2013 ▸; Cambridge Structural Database refcode NIKROQ).

**Figure 1:**
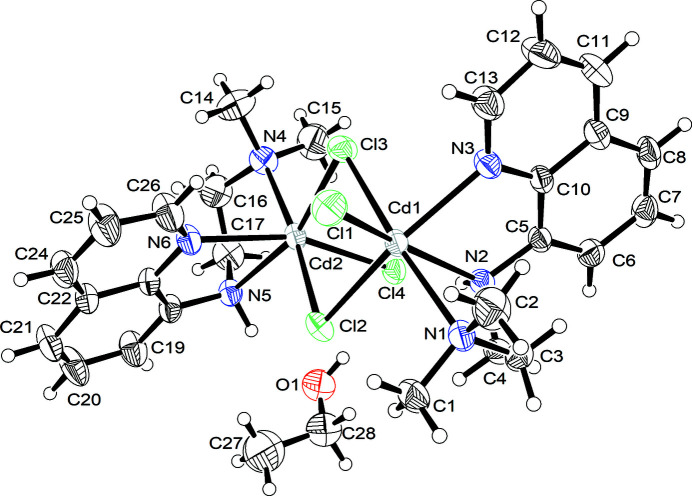
The mol­ecular structure of **I** showing 50% displacement ellipsoids.

**Figure 2:**
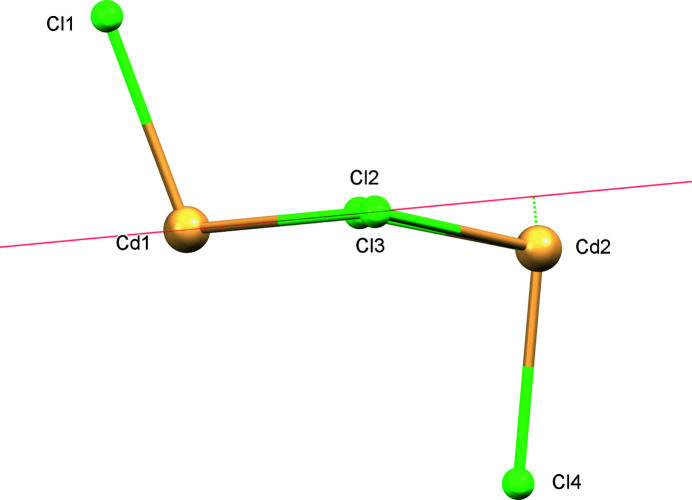
Detail of a Cl–(Cd_2_Cl_2_)–Cl fragment of **I** showing the deviation of Cd2 from the plane of Cl2, Cd1 and Cl3 as a green dotted line.

The asymmetric unit of **I** (Fig. 1 ▸) comprises one bimetallic complex unit and an ethanol solvent mol­ecule, implying the dinuclear molecules lacks crystallographic symmetry. Unlike the hydrate form of the complex (Al-Sudani & Kariuki, 2013 ▸), the Cd_2_Cl_2_ core in **I** is not strictly planar. One Cd^2+^ ion deviates by 0.565 (1) Å from the plane of the other Cd^2+^ and two Cl^−^ ions of the core (Fig. 2 ▸). The Cd1⋯Cd2 separation is 3.8061 (4) Å. The two pendant Cl^−^ ions are oriented roughly perpendicular to, but on opposite sides, of the plane of the (Cd_2_Cl_2_) core in both the hydrate and ethanol solvate forms. Similar perpendicular arrangement of the pendant Cl^−^ ions is observed in the Cl–(Cd_2_Cl_2_)–Cl fragments of other complexes with different ligands (Neis *et al.*, 2010 ▸; Marsh 1999 ▸; Pauly *et al.*, 2000 ▸). An alternative co-planar arrangement is also possible (Cannas *et al.*, 1980 ▸).

Both Cd^2+^ ions in **I** are coordinated by six atoms in a distorted octa­hedral geometry: three of the contacts are nitro­gen atoms from a tridentate ligand and the rest are chloride ions. Distortions in the coordination from ideal 90° angles range from 71.48 (9)° (N3—Cd1—N2) to 105.73 (3)° (Cl1—Cd1–Cl2) for one Cd^2+^ ion and 71.04 (9) ° (N6—Cd2—N5) to 102.09 (7)° (N5—Cd2—Cl2) for the other. The corres­ponding angles for the hydrate structure are in the range 69.48 (5) to 101.08 (4)°. The N—C—C—N torsion angles in the ethane di­amine are almost the same for both independent ligands [N1—C3—C4—N2 = 63.0 (4)° and N4—C16—C17—N5 = 63.3 (5)°] in **I**.

**Figure 3:**
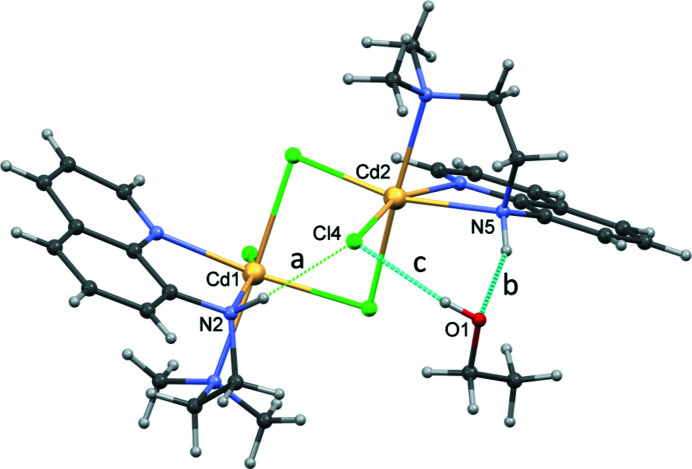
The asymmetric unit of **I** showing the intra­molecular contact (*a*) and hydrogen bonding with the ethanol solvent mol­ecule (*b* and *c*).

**Figure 4:**
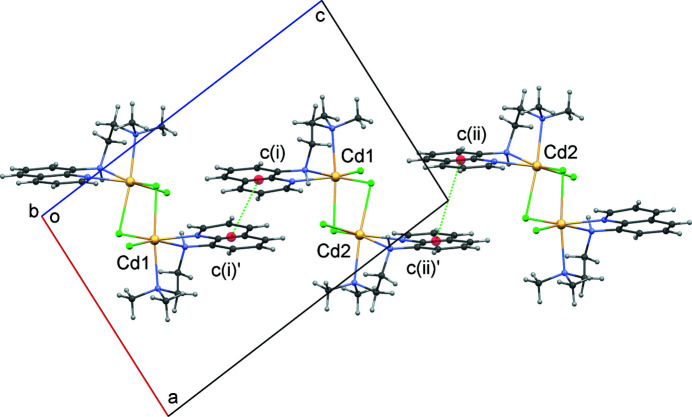
A segment of the crystal structure viewed down the *b* axis showing centroid–centroid contacts *c*(i)⋯*c*(i)′ and *c*(ii)⋯*c*(ii)′ for inversion symmetry related quinoline ring systems (C5–C13/N3) and (C18–C26/N6), respectively.

An intra­molecular N—H⋯Cl hydrogen bond (Table 1 ▸, Fig. 3 ▸) is observed in the dinuclear molecule. The complex also donates an N—H⋯O hydrogen bond to the ethanol solvent mol­ecule and accepts an O—H⋯Cl contact from the same mol­ecule to generate an *R*(6)^2^_2_ loop. In the extended structure, the quinoline ring systems of neighbouring complex units are involved in weak aromatic π–π stacking inter­actions. The groups involved are related by inversion symmetry with a *c*(i)⋯*c*(i)′ separation of 3.93 (1) Å [*c*(i) = the midpoint of the C9—C10 bond of the C5–C13/N3 ring system]. If a second longer inversion-related contact *c*(ii)⋯*c*(ii)′ of 4.56 (1) Å [*c*(ii) = midpoint of the C22—C23 bond of the C18–C26/N6 ring system] is considered to be a significant inter­action, infinite chains running parallel to [101] result (Fig. 4 ▸).

## Synthesis and crystallization

The 8-[2-(dimethyl­amino)­ethyl­amino]­quinoline ligand and cadmium dichloride were mixed in dry ethanol solvent at room temperature under a positive nitro­gen pressure and the mixture was stirred at room temperature for several hours. The solution was then warmed to dissolve the material and the product was recrystallized on cooling to produce colourless crystals of **I**.

## Refinement

Crystal data, data collection and structure refinement details are summarized in Table 2 ▸.

## Supplementary Material

Crystal structure: contains datablock(s) I. DOI: 10.1107/S2414314621001504/hb4375sup1.cif

Structure factors: contains datablock(s) I. DOI: 10.1107/S2414314621001504/hb4375Isup2.hkl

CCDC reference: 2062006

Additional supporting information:  crystallographic information; 3D view; checkCIF report

## Figures and Tables

**Table 1:** Hydrogen-bond geometry (Å, °)

*D*—H⋯*A*	*D*—H	H⋯*A*	*D*⋯*A*	*D*—H⋯*A*
N2—H2⋯Cl4	0.98	2.53	3.492 (3)	166
N5—H5⋯O1	0.98	1.94	2.874 (4)	158
O1—H1⋯Cl4	0.82	2.33	3.136 (3)	166

**Table 2:** Experimental details

Crystal data	
Chemical formula	[Cd_2_Cl_4_(C_13_H_17_N_3_)_2_]·C_2_H_6_O
*M*_r_	843.26
Crystal system, space group	Monoclinic, *P*2_1_/*c*
Temperature (K)	296
*a*, *b*, *c* (Å)	11.9747 (6), 15.6483 (7), 17.8804 (8)
β (°)	95.292 (4)
*V* (Å^3^)	3336.2 (3)
*Z*	4
Radiation type	Mo *K*α
μ (mm^−1^)	1.63
Crystal size (mm)	0.16 × 0.13 × 0.10

Data collection	
Diffractometer	Rigaku Oxford Diffraction SuperNova, Dual, Cu at home/near, Atlas
Absorption correction	Gaussian (*CrysAlis PRO* (Rigaku OD, 2019 ▸)
*T*_min_, *T*_max_	0.897, 1.000
No. of measured, independent and observed [*I* > 2σ(*I*)] reflections	29581, 8400, 5798
*R*_int_	0.033
(sin θ/λ)_max_ (Å^−1^)	0.700

Refinement	
*R*[*F* ^2^ > 2σ(*F* ^2^)], *wR*(*F* ^2^), *S*	0.036, 0.086, 1.05
No. of reflections	8400
No. of parameters	376
H-atom treatment	H-atom parameters constrained
Δρ_max_, Δρ_min_ (e Å^−3^)	0.58, −0.86

Computer programs: *CrysAlis PRO* (Rigaku OD, 2019 ▸), *SHELXS* (Sheldrick, 2008 ▸), *SHELXL* (Sheldrick, 2015 ▸), *Mercury* (Macrae *et al.*, 2020 ▸) and *WinGX* (Farrugia, 2012 ▸).

## full crystallographic data

### Crystal data

**Table:**

[Cd_2_Cl_4_(C_13_H_17_N_3_)_2_]·C_2_H_6_O	*F*(000) = 1688
*M**_r_* = 843.26	*D*_x_ = 1.679 Mg m^−^^3^
Monoclinic, *P*2_1_/*c*	Mo *K*α radiation, λ = 0.71073 Å
*a* = 11.9747 (6) Å	Cell parameters from 7578 reflections
*b* = 15.6483 (7) Å	θ = 3.7–29.2°
*c* = 17.8804 (8) Å	µ = 1.63 mm^−^^1^
β = 95.292 (4)°	*T* = 296 K
*V* = 3336.2 (3) Å^3^	Block, colourless
*Z* = 4	0.16 × 0.13 × 0.10 mm

### Data collection

**Table:**

Rigaku Oxford Diffraction SuperNova, Dual, Cu at home/near, Atlas diffractometer	5798 reflections with *I* > 2σ(*I*)
ω scans	*R*_int_ = 0.033
Absorption correction: gaussian (CrysAlisPro (Rigaku OD, 2019)	θ_max_ = 29.9°, θ_min_ = 1.7°
*T*_min_ = 0.897, *T*_max_ = 1.000	*h* = −16→15
29581 measured reflections	*k* = −21→20
8400 independent reflections	*l* = −23→24

### Refinement

**Table:**

Refinement on *F*^2^	0 restraints
Least-squares matrix: full	Hydrogen site location: inferred from neighbouring sites
*R*[*F*^2^ > 2σ(*F*^2^)] = 0.036	H-atom parameters constrained
*wR*(*F*^2^) = 0.086	*w* = 1/[σ^2^(*F*_o_^2^) + (0.0253*P*)^2^ + 3.3741*P*] where *P* = (*F*_o_^2^ + 2*F*_c_^2^)/3
*S* = 1.05	(Δ/σ)_max_ = 0.002
8400 reflections	Δρ_max_ = 0.58 e Å^−^^3^
376 parameters	Δρ_min_ = −0.86 e Å^−^^3^

### Special details

**Table:**

Geometry. All esds (except the esd in the dihedral angle between two l.s. planes) are estimated using the full covariance matrix. The cell esds are taken into account individually in the estimation of esds in distances, angles and torsion angles; correlations between esds in cell parameters are only used when they are defined by crystal symmetry. An approximate (isotropic) treatment of cell esds is used for estimating esds involving l.s. planes.
Refinement. The H atoms were positioned geometrically and refined using a riding model with *U*_iso_(H) = 1.2*U*_eq_(carrier) or 1.5*U*_eq_(methyl C). The methyl groups were allowd to rotate, but not to tip, to best fit the electron density.

### Fractional atomic coordinates and isotropic or equivalent isotropic displacement parameters (Å^2^)

**Table:**

	*x*	*y*	*z*	*U*_iso_*/*U*_eq_	
C1	0.5109 (3)	0.4794 (3)	0.09136 (19)	0.0610 (11)	
H1A	0.468166	0.531360	0.091253	0.092*	
H1B	0.466152	0.435355	0.066115	0.092*	
H1C	0.576927	0.488698	0.065837	0.092*	
C2	0.6062 (4)	0.3720 (3)	0.1695 (3)	0.0719 (13)	
H2A	0.673719	0.380147	0.145320	0.108*	
H2B	0.560683	0.329261	0.142997	0.108*	
H2C	0.624854	0.353891	0.220385	0.108*	
C3	0.6125 (3)	0.5191 (3)	0.2093 (2)	0.0579 (11)	
H3A	0.661288	0.544847	0.175199	0.069*	
H3B	0.659558	0.492550	0.249900	0.069*	
C4	0.5426 (3)	0.5879 (2)	0.2411 (2)	0.0531 (10)	
H4A	0.591226	0.631940	0.264266	0.064*	
H4B	0.494231	0.613984	0.200942	0.064*	
C5	0.5393 (3)	0.5251 (2)	0.36625 (17)	0.0358 (7)	
C6	0.6046 (3)	0.5815 (3)	0.40930 (19)	0.0453 (9)	
H6	0.609177	0.638054	0.393875	0.054*	
C7	0.6649 (3)	0.5551 (3)	0.4766 (2)	0.0516 (10)	
H7	0.709823	0.594159	0.504751	0.062*	
C8	0.6582 (3)	0.4734 (3)	0.50079 (19)	0.0486 (9)	
H8	0.697695	0.456832	0.545688	0.058*	
C9	0.5915 (3)	0.4130 (2)	0.45811 (18)	0.0432 (9)	
C10	0.5328 (3)	0.4391 (2)	0.38922 (17)	0.0360 (7)	
C11	0.5772 (4)	0.3284 (3)	0.4815 (2)	0.0594 (11)	
H11	0.613726	0.309528	0.526641	0.071*	
C12	0.5104 (4)	0.2742 (3)	0.4385 (2)	0.0670 (12)	
H12	0.498995	0.218474	0.454222	0.080*	
C13	0.4587 (3)	0.3039 (3)	0.3697 (2)	0.0562 (10)	
H13	0.415285	0.265768	0.339554	0.067*	
C14	−0.0799 (4)	0.4866 (3)	0.3591 (3)	0.0781 (14)	
H14A	−0.023902	0.450126	0.384180	0.117*	
H14B	−0.103480	0.463456	0.310456	0.117*	
H14C	−0.143177	0.490197	0.388244	0.117*	
C15	0.0095 (4)	0.6041 (4)	0.4240 (2)	0.0832 (15)	
H15A	0.039218	0.660568	0.419081	0.125*	
H15B	0.067710	0.566875	0.445384	0.125*	
H15C	−0.050415	0.605741	0.456112	0.125*	
C16	−0.1216 (3)	0.6266 (3)	0.3123 (3)	0.0659 (12)	
H16A	−0.166148	0.592189	0.275586	0.079*	
H16B	−0.170664	0.645949	0.349116	0.079*	
C17	−0.0792 (3)	0.7015 (3)	0.2743 (2)	0.0576 (10)	
H17A	−0.033743	0.735832	0.310602	0.069*	
H17B	−0.141975	0.736112	0.254028	0.069*	
C18	−0.0760 (3)	0.6499 (2)	0.14515 (19)	0.0409 (8)	
C19	−0.1360 (3)	0.7065 (3)	0.0994 (2)	0.0609 (11)	
H19	−0.137400	0.763865	0.112889	0.073*	
C20	−0.1960 (4)	0.6794 (3)	0.0321 (2)	0.0741 (14)	
H20	−0.236205	0.719086	0.001762	0.089*	
C21	−0.1958 (4)	0.5963 (3)	0.0110 (2)	0.0626 (12)	
H21	−0.235248	0.579364	−0.033762	0.075*	
C22	−0.1355 (3)	0.5352 (2)	0.05715 (19)	0.0434 (8)	
C23	−0.0746 (3)	0.5624 (2)	0.12458 (18)	0.0370 (7)	
C24	−0.1319 (4)	0.4480 (3)	0.0387 (2)	0.0594 (11)	
H24	−0.171148	0.427885	−0.005102	0.071*	
C25	−0.0715 (4)	0.3937 (3)	0.0846 (3)	0.0722 (13)	
H25	−0.069524	0.335704	0.073334	0.087*	
C26	−0.0121 (4)	0.4254 (3)	0.1492 (2)	0.0655 (12)	
H26	0.030561	0.387245	0.179747	0.079*	
C27	0.1593 (5)	0.7698 (4)	0.0786 (3)	0.112 (2)	
H27A	0.127708	0.713794	0.082293	0.168*	
H27B	0.207118	0.770868	0.038332	0.168*	
H27C	0.100097	0.810805	0.068762	0.168*	
C28	0.2249 (4)	0.7913 (3)	0.1491 (3)	0.0789 (14)	
H28A	0.277926	0.745579	0.162022	0.095*	
H28B	0.267414	0.842890	0.141981	0.095*	
N1	0.5435 (2)	0.4533 (2)	0.16910 (16)	0.0465 (7)	
N2	0.4735 (2)	0.55133 (17)	0.29757 (14)	0.0367 (6)	
H2	0.419375	0.594663	0.310394	0.044*	
N3	0.4685 (2)	0.38266 (18)	0.34557 (15)	0.0415 (7)	
N4	−0.0328 (3)	0.5724 (2)	0.35048 (17)	0.0490 (8)	
N5	−0.0111 (2)	0.67654 (17)	0.21276 (15)	0.0393 (6)	
H5	0.033935	0.726058	0.200603	0.047*	
N6	−0.0127 (2)	0.50655 (18)	0.16967 (16)	0.0434 (7)	
Cd1	0.37193 (2)	0.43475 (2)	0.23492 (2)	0.03457 (7)	
Cd2	0.11394 (2)	0.56817 (2)	0.26458 (2)	0.03612 (8)	
Cl1	0.32762 (8)	0.30005 (6)	0.16560 (5)	0.0542 (2)	
Cl2	0.25905 (7)	0.55385 (6)	0.15909 (5)	0.0450 (2)	
Cl3	0.19490 (7)	0.42895 (6)	0.32090 (5)	0.0450 (2)	
Cl4	0.24434 (8)	0.67656 (6)	0.33611 (5)	0.0487 (2)	
O1	0.1615 (3)	0.8033 (2)	0.20690 (19)	0.0813 (9)	
H1	0.189522	0.777756	0.244020	0.122*	

### Atomic displacement parameters (Å^2^)

**Table:**

	*U*^11^	*U*^22^	*U*^33^	*U*^12^	*U*^13^	*U*^23^
C1	0.056 (2)	0.094 (3)	0.032 (2)	−0.003 (2)	−0.0001 (17)	0.005 (2)
C2	0.052 (3)	0.088 (4)	0.076 (3)	0.021 (2)	0.012 (2)	0.004 (3)
C3	0.040 (2)	0.096 (3)	0.037 (2)	−0.020 (2)	0.0032 (16)	0.006 (2)
C4	0.064 (3)	0.053 (2)	0.039 (2)	−0.024 (2)	−0.0120 (17)	0.0115 (18)
C5	0.0321 (17)	0.046 (2)	0.0283 (16)	0.0012 (15)	0.0003 (13)	0.0006 (15)
C6	0.044 (2)	0.053 (2)	0.0384 (19)	−0.0063 (17)	0.0016 (15)	−0.0013 (17)
C7	0.043 (2)	0.074 (3)	0.037 (2)	−0.0076 (19)	−0.0017 (15)	−0.0125 (19)
C8	0.039 (2)	0.074 (3)	0.0306 (18)	0.0038 (19)	−0.0061 (14)	−0.0032 (19)
C9	0.0387 (19)	0.058 (2)	0.0330 (18)	0.0123 (17)	0.0006 (14)	0.0030 (17)
C10	0.0304 (16)	0.045 (2)	0.0320 (17)	0.0055 (15)	−0.0006 (12)	0.0019 (15)
C11	0.069 (3)	0.064 (3)	0.043 (2)	0.014 (2)	−0.0100 (18)	0.014 (2)
C12	0.091 (3)	0.050 (3)	0.058 (3)	0.005 (2)	−0.006 (2)	0.018 (2)
C13	0.066 (3)	0.044 (2)	0.056 (2)	0.000 (2)	−0.0108 (19)	0.0013 (19)
C14	0.074 (3)	0.073 (3)	0.091 (4)	−0.016 (3)	0.030 (3)	0.008 (3)
C15	0.083 (3)	0.113 (4)	0.054 (3)	−0.012 (3)	0.012 (2)	−0.012 (3)
C16	0.051 (2)	0.075 (3)	0.073 (3)	0.013 (2)	0.014 (2)	0.003 (2)
C17	0.063 (3)	0.049 (2)	0.061 (2)	0.016 (2)	0.008 (2)	−0.008 (2)
C18	0.0350 (18)	0.039 (2)	0.047 (2)	0.0029 (15)	−0.0057 (15)	−0.0014 (16)
C19	0.069 (3)	0.041 (2)	0.067 (3)	0.012 (2)	−0.020 (2)	−0.004 (2)
C20	0.085 (3)	0.066 (3)	0.064 (3)	0.024 (3)	−0.032 (2)	0.005 (2)
C21	0.067 (3)	0.066 (3)	0.050 (2)	0.009 (2)	−0.0244 (19)	−0.002 (2)
C22	0.0394 (19)	0.048 (2)	0.0405 (19)	−0.0004 (16)	−0.0075 (15)	−0.0035 (17)
C23	0.0293 (16)	0.042 (2)	0.0380 (18)	0.0012 (14)	−0.0040 (13)	−0.0002 (15)
C24	0.070 (3)	0.055 (3)	0.049 (2)	−0.006 (2)	−0.0152 (19)	−0.014 (2)
C25	0.098 (4)	0.041 (2)	0.072 (3)	0.003 (2)	−0.029 (3)	−0.015 (2)
C26	0.085 (3)	0.040 (2)	0.066 (3)	0.008 (2)	−0.025 (2)	−0.005 (2)
C27	0.116 (5)	0.124 (6)	0.096 (4)	−0.015 (4)	0.008 (4)	−0.006 (4)
C28	0.080 (3)	0.079 (4)	0.077 (3)	−0.006 (3)	0.005 (3)	0.008 (3)
N1	0.0359 (16)	0.064 (2)	0.0386 (16)	0.0012 (14)	−0.0001 (12)	0.0056 (15)
N2	0.0389 (15)	0.0333 (15)	0.0370 (15)	−0.0010 (12)	−0.0011 (11)	0.0032 (12)
N3	0.0457 (17)	0.0372 (17)	0.0401 (16)	0.0039 (13)	−0.0053 (12)	0.0035 (13)
N4	0.0506 (18)	0.051 (2)	0.0455 (18)	0.0058 (15)	0.0060 (14)	0.0003 (15)
N5	0.0423 (16)	0.0299 (15)	0.0442 (16)	0.0006 (12)	−0.0046 (12)	−0.0035 (13)
N6	0.0494 (18)	0.0318 (16)	0.0458 (17)	0.0027 (13)	−0.0123 (13)	−0.0022 (13)
Cd1	0.03461 (13)	0.03321 (14)	0.03431 (13)	−0.00004 (10)	−0.00531 (9)	0.00115 (10)
Cd2	0.03520 (14)	0.03407 (14)	0.03719 (14)	0.00278 (10)	−0.00686 (10)	−0.00189 (11)
Cl1	0.0645 (6)	0.0397 (5)	0.0574 (6)	−0.0067 (4)	0.0004 (4)	−0.0091 (4)
Cl2	0.0485 (5)	0.0479 (5)	0.0370 (4)	0.0107 (4)	−0.0046 (4)	0.0047 (4)
Cl3	0.0452 (5)	0.0395 (5)	0.0501 (5)	0.0074 (4)	0.0039 (4)	0.0100 (4)
Cl4	0.0523 (5)	0.0419 (5)	0.0483 (5)	−0.0069 (4)	−0.0145 (4)	−0.0010 (4)
O1	0.080 (2)	0.079 (2)	0.082 (2)	−0.0113 (19)	−0.0056 (18)	0.0220 (19)

### Geometric parameters (Å, º)

**Table:**

C1—N1	1.467 (4)	C16—H16B	0.9700
C1—H1A	0.9600	C17—N5	1.481 (4)
C1—H1B	0.9600	C17—H17A	0.9700
C1—H1C	0.9600	C17—H17B	0.9700
C2—N1	1.476 (5)	C18—C19	1.364 (5)
C2—H2A	0.9600	C18—C23	1.418 (5)
C2—H2B	0.9600	C18—N5	1.437 (4)
C2—H2C	0.9600	C19—C20	1.409 (5)
C3—N1	1.467 (5)	C19—H19	0.9300
C3—C4	1.508 (6)	C20—C21	1.354 (6)
C3—H3A	0.9700	C20—H20	0.9300
C3—H3B	0.9700	C21—C22	1.417 (5)
C4—N2	1.478 (4)	C21—H21	0.9300
C4—H4A	0.9700	C22—C24	1.405 (5)
C4—H4B	0.9700	C22—C23	1.416 (4)
C5—C6	1.368 (5)	C23—N6	1.361 (4)
C5—C10	1.412 (5)	C24—C25	1.345 (6)
C5—N2	1.455 (4)	C24—H24	0.9300
C6—C7	1.407 (5)	C25—C26	1.390 (5)
C6—H6	0.9300	C25—H25	0.9300
C7—C8	1.356 (6)	C26—N6	1.322 (5)
C7—H7	0.9300	C26—H26	0.9300
C8—C9	1.414 (5)	C27—C28	1.462 (7)
C8—H8	0.9300	C27—H27A	0.9600
C9—C11	1.404 (5)	C27—H27B	0.9600
C9—C10	1.421 (4)	C27—H27C	0.9600
C10—N3	1.368 (4)	C28—O1	1.350 (5)
C11—C12	1.356 (6)	C28—H28A	0.9700
C11—H11	0.9300	C28—H28B	0.9700
C12—C13	1.404 (5)	N1—Cd1	2.477 (3)
C12—H12	0.9300	N2—Cd1	2.411 (3)
C13—N3	1.315 (5)	N2—H2	0.9800
C13—H13	0.9300	N3—Cd1	2.344 (3)
C14—N4	1.469 (5)	N4—Cd2	2.439 (3)
C14—H14A	0.9600	N5—Cd2	2.392 (3)
C14—H14B	0.9600	N5—H5	0.9800
C14—H14C	0.9600	N6—Cd2	2.374 (3)
C15—N4	1.452 (5)	Cd1—Cl1	2.4777 (9)
C15—H15A	0.9600	Cd1—Cl2	2.6079 (9)
C15—H15B	0.9600	Cd1—Cl3	2.7326 (9)
C15—H15C	0.9600	Cd2—Cl3	2.5535 (9)
C16—C17	1.468 (6)	Cd2—Cl4	2.5656 (9)
C16—N4	1.478 (5)	Cd2—Cl2	2.6893 (9)
C16—H16A	0.9700	O1—H1	0.8200
			
N1—C1—H1A	109.5	C23—C22—C21	119.1 (3)
N1—C1—H1B	109.5	N6—C23—C22	121.3 (3)
H1A—C1—H1B	109.5	N6—C23—C18	119.0 (3)
N1—C1—H1C	109.5	C22—C23—C18	119.6 (3)
H1A—C1—H1C	109.5	C25—C24—C22	119.8 (4)
H1B—C1—H1C	109.5	C25—C24—H24	120.1
N1—C2—H2A	109.5	C22—C24—H24	120.1
N1—C2—H2B	109.5	C24—C25—C26	119.1 (4)
H2A—C2—H2B	109.5	C24—C25—H25	120.5
N1—C2—H2C	109.5	C26—C25—H25	120.5
H2A—C2—H2C	109.5	N6—C26—C25	123.9 (4)
H2B—C2—H2C	109.5	N6—C26—H26	118.1
N1—C3—C4	112.3 (3)	C25—C26—H26	118.1
N1—C3—H3A	109.2	C28—C27—H27A	109.5
C4—C3—H3A	109.2	C28—C27—H27B	109.5
N1—C3—H3B	109.2	H27A—C27—H27B	109.5
C4—C3—H3B	109.2	C28—C27—H27C	109.5
H3A—C3—H3B	107.9	H27A—C27—H27C	109.5
N2—C4—C3	110.3 (3)	H27B—C27—H27C	109.5
N2—C4—H4A	109.6	O1—C28—C27	113.4 (5)
C3—C4—H4A	109.6	O1—C28—H28A	108.9
N2—C4—H4B	109.6	C27—C28—H28A	108.9
C3—C4—H4B	109.6	O1—C28—H28B	108.9
H4A—C4—H4B	108.1	C27—C28—H28B	108.9
C6—C5—C10	119.7 (3)	H28A—C28—H28B	107.7
C6—C5—N2	122.0 (3)	C3—N1—C1	110.9 (3)
C10—C5—N2	118.3 (3)	C3—N1—C2	109.8 (3)
C5—C6—C7	120.9 (4)	C1—N1—C2	109.6 (3)
C5—C6—H6	119.5	C3—N1—Cd1	107.7 (2)
C7—C6—H6	119.5	C1—N1—Cd1	108.8 (2)
C8—C7—C6	120.6 (4)	C2—N1—Cd1	109.9 (2)
C8—C7—H7	119.7	C5—N2—C4	113.1 (3)
C6—C7—H7	119.7	C5—N2—Cd1	112.9 (2)
C7—C8—C9	120.4 (3)	C4—N2—Cd1	105.4 (2)
C7—C8—H8	119.8	C5—N2—H2	108.4
C9—C8—H8	119.8	C4—N2—H2	108.4
C11—C9—C8	123.2 (3)	Cd1—N2—H2	108.4
C11—C9—C10	117.7 (3)	C13—N3—C10	118.7 (3)
C8—C9—C10	119.1 (4)	C13—N3—Cd1	123.4 (2)
N3—C10—C5	119.5 (3)	C10—N3—Cd1	117.7 (2)
N3—C10—C9	121.1 (3)	C15—N4—C14	108.8 (4)
C5—C10—C9	119.3 (3)	C15—N4—C16	113.4 (4)
C12—C11—C9	120.2 (4)	C14—N4—C16	107.8 (3)
C12—C11—H11	119.9	C15—N4—Cd2	111.6 (3)
C9—C11—H11	119.9	C14—N4—Cd2	110.3 (2)
C11—C12—C13	118.6 (4)	C16—N4—Cd2	104.7 (2)
C11—C12—H12	120.7	C18—N5—C17	114.2 (3)
C13—C12—H12	120.7	C18—N5—Cd2	113.0 (2)
N3—C13—C12	123.5 (4)	C17—N5—Cd2	105.5 (2)
N3—C13—H13	118.2	C18—N5—H5	108.0
C12—C13—H13	118.2	C17—N5—H5	108.0
N4—C14—H14A	109.5	Cd2—N5—H5	108.0
N4—C14—H14B	109.5	C26—N6—C23	118.0 (3)
H14A—C14—H14B	109.5	C26—N6—Cd2	124.7 (2)
N4—C14—H14C	109.5	C23—N6—Cd2	116.1 (2)
H14A—C14—H14C	109.5	N3—Cd1—N2	71.48 (9)
H14B—C14—H14C	109.5	N3—Cd1—N1	94.32 (10)
N4—C15—H15A	109.5	N2—Cd1—N1	74.16 (10)
N4—C15—H15B	109.5	N3—Cd1—Cl1	101.11 (7)
H15A—C15—H15B	109.5	N2—Cd1—Cl1	162.14 (7)
N4—C15—H15C	109.5	N1—Cd1—Cl1	90.61 (8)
H15A—C15—H15C	109.5	N3—Cd1—Cl2	151.53 (7)
H15B—C15—H15C	109.5	N2—Cd1—Cl2	85.18 (7)
C17—C16—N4	114.0 (3)	N1—Cd1—Cl2	94.71 (7)
C17—C16—H16A	108.8	Cl1—Cd1—Cl2	105.73 (3)
N4—C16—H16A	108.8	N3—Cd1—Cl3	82.05 (7)
C17—C16—H16B	108.8	N2—Cd1—Cl3	98.52 (7)
N4—C16—H16B	108.8	N1—Cd1—Cl3	172.59 (7)
H16A—C16—H16B	107.6	Cl1—Cd1—Cl3	96.41 (3)
C16—C17—N5	111.8 (3)	Cl2—Cd1—Cl3	85.67 (3)
C16—C17—H17A	109.3	N6—Cd2—N5	71.04 (9)
N5—C17—H17A	109.3	N6—Cd2—N4	90.64 (10)
C16—C17—H17B	109.3	N5—Cd2—N4	76.30 (10)
N5—C17—H17B	109.3	N6—Cd2—Cl3	97.45 (7)
H17A—C17—H17B	107.9	N5—Cd2—Cl3	163.54 (7)
C19—C18—C23	119.3 (3)	N4—Cd2—Cl3	92.51 (8)
C19—C18—N5	122.0 (3)	N6—Cd2—Cl4	160.78 (7)
C23—C18—N5	118.7 (3)	N5—Cd2—Cl4	93.15 (7)
C18—C19—C20	121.0 (4)	N4—Cd2—Cl4	96.26 (8)
C18—C19—H19	119.5	Cl3—Cd2—Cl4	100.13 (3)
C20—C19—H19	119.5	N6—Cd2—Cl2	82.90 (8)
C21—C20—C19	120.9 (4)	N5—Cd2—Cl2	102.09 (7)
C21—C20—H20	119.5	N4—Cd2—Cl2	173.50 (8)
C19—C20—H20	119.5	Cl3—Cd2—Cl2	87.66 (3)
C20—C21—C22	120.0 (4)	Cl4—Cd2—Cl2	90.11 (3)
C20—C21—H21	120.0	Cd1—Cl2—Cd2	91.85 (3)
C22—C21—H21	120.0	Cd2—Cl3—Cd1	92.05 (3)
C24—C22—C23	117.9 (3)	C28—O1—H1	109.5
C24—C22—C21	123.0 (3)		

### Hydrogen-bond geometry (Å, º)

**Table:**

*D*—H···*A*	*D*—H	H···*A*	*D*···*A*	*D*—H···*A*
N2—H2···Cl4	0.98	2.53	3.492 (3)	166
N5—H5···O1	0.98	1.94	2.874 (4)	158
O1—H1···Cl4	0.82	2.33	3.136 (3)	166
